# Infographic: towards evidence-based simulation training

**DOI:** 10.31744/einstein_journal/2026CE2393

**Published:** 2026-03-27

**Authors:** Thomaz Bittencourt Couto, Alessandra Mazzo, Brena Carvalho Pinto de Melo, Mariana Santos Alecrim Molina, Priscilla Cerullo Hashimoto, Joyce Kelly Barreto Silva, Desiree Gonçalves, Dario Cecilio-Fernandes

**Affiliations:** 1 Hospital Israelita Albert Einstein São Paulo SP Brazil Hospital Israelita Albert Einstein, São Paulo, SP, Brazil.; 2 Universidade de São Paulo Bauru SP Brazil Universidade de São Paulo, Bauru, SP, Brazil.; 3 Faculdade Pernambucana de Saúde Recife PE Brazil Faculdade Pernambucana de Saúde, Recife, PE, Brazil.; 4 Erasmus MC University Medical Centre Rotterdam Institute of Medical Education Research Rotterdam Rotterdam Netherlands Institute of Medical Education Research Rotterdam, Erasmus MC University Medical Centre Rotterdam, Rotterdam, The Netherlands.

Dear Editor,

Following the publication of our editorial on evidence-based approaches to simulation-based education (SBE),^(1)^ we received multiple requests from educators and the simulation community for a concise resource to support implementation at the point of curriculum design and faculty training. To address this gap, we created a one-page infographic that translates the editorial's arguments into a practical visual aid (Figure 1).

**Figure 1:**
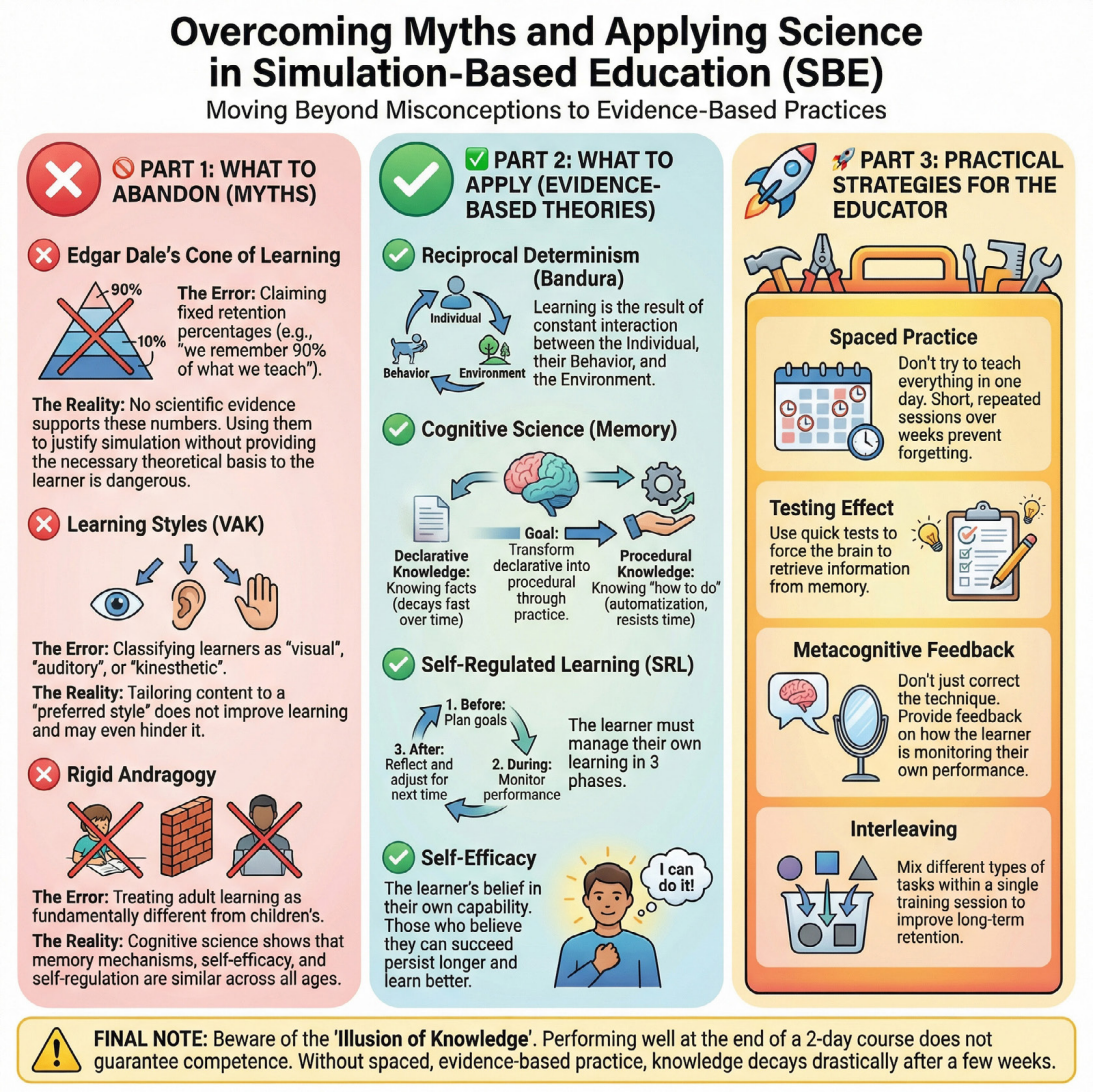
Infographic: towards evidence-based simulation training

Source: created with Nano Banana Pro, based on Cecilio-Fernandes et al.^(1)^

Infographics are a tool for communicating information by combining text and visual elements to present complex concepts in an accessible and memorable format. They reduce cognitive load, enhance comprehension, and improve long-term retention compared with text-only materials, making them valuable for faculty and students.^(2)^ Their visual structure facilitates dissemination and visibility of research findings across digital platforms, where they are shared significantly more frequently than textual summaries.^(3)^

Creating an infographic uses the cognitive principles discussed in the editorial itself, as visual summaries support retrieval cues and improve knowledge organization in ways that are congruent with findings from cognitive science. The visual consolidates our paper's arguments into a single frame that can act as a talking map for abandoning myths while adopting theory-driven practices. This figure repackages the published synthesis into an implementation-ready format that facilitates learning.

By pairing the editorial with an infographic, the journal can enhance the translational impact of the piece, supporting educators who aim to phase out myth-based practices while adopting theory-guided strategies that are more likely to yield durable learning in simulation based education.

On behalf of all authors,

## Data Availability

The underlying content is contained within the manuscript.
